# Evaluating an adapted reverse categorisation task to assess cognitive flexibility in young children with Down syndrome

**DOI:** 10.1111/jir.13040

**Published:** 2023-05-23

**Authors:** K. Van Deusen, M. A. Prince, A. J. Thurman, A. J. Esbensen, L. R. Patel, L. Abbeduto, M. M. Walsh, L. A. Daunhauer, R. T. Feigles, D. J. Fidler

**Affiliations:** 1 Human Development and Family Studies, Colorado State University, Fort Collins, CO, USA; 2 MIND Institute, University of California Davis, Sacramento, CA, USA; 3 Department of Psychiatry and Behavioral Sciences, University of California Davis Health, Sacramento, CA, USA; 4 Division of Developmental and Behavioral Pediatrics, Cincinnati Children’s Hospital Medical Center, Cincinnati, OH, USA; 5 College of Medicine, University of Cincinnati, Cincinnati, OH, USA; 6 Department of Psychiatry, University of Colorado Anschutz Medical Campus, Aurora, CO, USA

**Keywords:** children, cognitive flexibility, Down syndrome, executive function, measurement

## Abstract

**Background:**

Accurate measurement of cognitive skills is necessary to advance both developmental and intervention science for individuals with Down syndrome (DS). This study evaluated the feasibility, developmental sensitivity and preliminary reliability of a reverse categorisation measure designed to assess cognitive flexibility in young children with DS.

**Methods:**

Seventy-two children with DS ages 2.5–8 years completed an adapted version of a reverse categorisation task. Twenty-eight of the participants were assessed again 2 weeks later for retest reliability.

**Results:**

This adapted measure demonstrated adequate feasibility and developmental sensitivity, and preliminary evidence for test–retest reliability when administered to children with DS in this age range.

**Conclusions:**

This adapted reverse categorisation measure may be useful for future developmental and treatment studies that target early foundations of cognitive flexibility in young children with DS.

## Background

Down syndrome (DS) is the most common neurogenetic syndrome associated with intellectual disability (ID; Presson *et al*. 2013) and predisposes individuals to cognitive regulation challenges, especially in the area of executive function (EF). EF refers to a collection of cognitive skills that are used when engaging in purposeful, goal-directed behaviour (Diamond 2013). Although there is ongoing discussion regarding the architecture of EF, most models include several core cognitive skills, including the dimension of *cognitive flexibility* (Miyake *et al*. 2000; Diamond 2006, 2013). Cognitive flexibility is the ability to adjust one’s thinking or behaviour in response to changes in information and task conditions (Miyake *et al*. 2000; Miyake & Friedman 2012; Laureys *et al*. 2022). This EF component is critical for goal pursuit because the ability to modify thinking or behaviour based on current circumstances enables one to adapt strategies and modify preset ideas when situational parameters change.

In the general population, cognitive flexibility has been linked to a range of adaptive outcomes, like academic foundations in math, phonemic awareness and letter knowledge (Blair & Razza 2007). In children with DS, cognitive flexibility has been linked to the development of language skills and adaptive behaviour (Landry *et al*. 2012; Will *et al*. 2021). Understanding the development of cognitive flexibility in children with DS can facilitate a more informed approach to EF intervention in this population. Findings to date suggest that flexibility in children with DS may be on par with children at similar developmental levels (Lee *et al*. 2011; Daunhauer *et al*. 2014; Loveall *et al*. 2017), with more pronounced challenges in this area during and after adolescence (Loveall *et al*. 2017; Onnivello *et al*. 2022).

One important limitation to our current knowledge base regarding cognitive flexibility in individuals with DS is that many of the available laboratory measures are appropriate only for individuals who have acquired relatively advanced cognitive–linguistic skills. Individuals with DS who demonstrate more pronounced overall cognitive delays are often excluded from participation in research studies using these measures and may be underrepresented in the literature (e.g. Landry *et al*. 2012; Schworer *et al*. 2023). This is particularly concerning in that individuals who demonstrate the most pronounced levels of delay in a given domain stand to potentially benefit the most from intervention.

## Measuring childhood cognitive flexibility

When studying cognitive flexibility in the general population, one common approach has been the use of set-shifting tasks, in which children are instructed to follow a specific rule and then the rule is changed, and children are instructed to follow the new rule (e.g. Object Classification Task for Children and Dimensional Change Card Sort task; Carlson 2005; Cassidy 2020; Perner & Lang 2002; Smidts *et al*. 2018). Cognitive flexibility is needed in these tasks to refrain from following the first rule learned and to shift to the use of the newer rule. Another approach involves reverse categorisation, wherein participants are instructed to sort blocks or animals by size (‘big’ and ‘little’) into corresponding big and little buckets (Carlson 2005). When the rule is switched, children are instructed to sort the little items into the big buckets and the big items into the little buckets (Carlson 2005).

Studies using these measures in the general population (Carlson 2005) have reported less flexibility at age 2 years, with increasing flexibility at age 3 years (Perner & Lang 2002; Brooks *et al*. 2003). However, the appropriateness of these measures has yet to be examined in children with developmental delays that result from neurogenetic conditions, such as DS. The presence of specific patterns of relative strength and challenge may confound performance on these tasks. Adaptations to avoid the influence of non-targeted skills are important for the valid measurement of cognitive flexibility skills and to avoid interpretational confounds.

## Use of early cognitive flexibility measures with children with Down syndrome

Although the measures described earlier require the use of cognitive flexibility, they also require non-executive skills for their successful completion, like receptive language (e.g. interpreting verbal instructions). In fact, verbal mental age (MA) has been shown to be associated with performance on flexibility tasks in adolescents and young adults with DS, which is notable in that verbal skills can often be an area of specific challenge within this population (Landry *et al*. 2012). These tasks often use two-dimensional objects like cards, requiring visual acuity and abstract representations, two areas of noted difficulty in individuals with DS (John *et al*. 2004; Fidler 2005). From an engagement perspective, card sorting tasks may elicit lower levels of child interest when compared with activities that involve toys and other manipulatives.

A growing interest in the psychometric evaluation of EF more broadly in DS has led to important new information regarding the feasibility and utility of widely used measures when administered to older children and adolescents (Schworer *et al*. 2021, 2022). These studies have taken a systematic approach to evaluating measures of EF that are routinely used in clinical practice to provide recommendations for using the most sensitive measures of developing working memory and social cognition in individuals with DS (Schworer *et al*. 2021, 2022). However, the assessment of EF in young children with DS is more challenging to evaluate because there are few standardised measures of EF during early childhood, and those that exist involve the use of non-targeted developmental skills for their successful completion. The identification of reliable and developmentally sensitive measurement tools will help to characterise the emergence of cognitive flexibility for children with DS and allow researchers to demonstrate therapeutic and intervention treatment effects for individuals with DS with accuracy (Esbensen *et al*. 2017).

## Current study

The present study was designed to examine the preliminary psychometric properties of an adapted reverse categorisation (ARC) measure of cognitive flexibility in 2.5- to 8-year-old children with DS. Adaptations to the task involved minimising receptive language and motor planning demands, the use of toys of high contrast in terms of visual appearance and shape, and enhancing motivation and engagement through the use of familiar referents. Task feasibility and developmental sensitivity were evaluated, and test–retest reliability was examined for a subset of participants.

## Methods

### Procedures

Participants were recruited into one of two projects focused on EF or EF assessment in young children with DS. Participation took place at several sites across the USA, including the West, Mountain West and Midwest. All procedures were approved by institutional review boards at each site. Caregiver and participant understanding of English was required for inclusion. Study data were collected and managed using REDCap electronic data capture tools hosted at Colorado State University (Harris *et al*. 2009, 2019).

Data collection coincided with the onset of the COVID-19 pandemic. As a result, 88.9% of participants (*n* = 64) were assessed with the use of safety precautions to prevent the spread of the COVID-19 virus (face masks, face shields/eye protection and/or scrubs).

### Participants

Participants were 72 children with DS who were 2.50–8.67 years old [Mean (M) = 5.22, standard deviation (SD) = 1.47]. The child participants were 44.9% male. The sample was predominantly White and non-Hispanic or Latino. Four caregivers did not complete questionnaires. For a complete description of participant demographic characteristics, see Table 1.

### Measures

#### Caregiver-reported measures

##### Medical history questionnaire.

Caregivers were asked to complete questions regarding their child’s medical history, including DS type, sensory difficulties (e.g. vision and hearing) and biomedical risk factors (e.g. prematurity).

#### Child developmental status

Mental age estimates were obtained for each child as follows. Participants 3 years and older were administered the Stanford–Binet 5th Edition Abbreviated Battery IQ (SB5-ABIQ; Roid 2003a). This measure was selected as the primary measure for the study to align with converging common data elements for DS assessment (Esbensen *et al*. 2017). However, because of the chronological age (CA) range of participants for this study, and the variability in developmental status among the children within this age range, a second measure, the Bayley Scales of Infant and Toddler Development 4th Edition (Bayley-4; Bayley & Alyward 2019), was administered to a subset of participants 2.50–4.99 years old to extend the MA equivalent estimations below the floor of the SB5-ABIQ. This additional administration took place in only one of the two projects. All children between the ages of 2.5 and 3 years completed only the Bayley-4 cognitive measure. The two measures are described in the succeeding text.

##### Stanford–Binet 5th Edition Abbreviated Battery IQ (Roid 2003a).

The SB5-ABIQ is a measurement tool for IQ in individuals 2.00 to 85.00 years old. For this study, child participants completed two subtests: Verbal Knowledge Vocabulary and Nonverbal Reasoning Object Series/Matrices. The ABIQ subtests have high internal consistency with the other scales in the SB5 (above 0.90; Roid 2003b). Raw scores were transformed into age equivalent scores in months. Trained graduate and professional research associates administered the SB5-ABIQ to participants 3 to 8 years old (*n* = 70).

##### Bayley Scales of Infant and Toddler Development 4th Edition (Bayley & Alyward 2019).

The Bayley-4 is a standardised assessment of cognition, communication and motor skills from 1 to 42 months (Bayley & Alyward 2019). The Bayley-4 cognitive scale has strong internal consistency overall (*r* = 0.95), including in children with DS (*r* = 0.98; Bayley & Alyward 2019). Trained graduate and professional research assistants administered the Bayley-4 cognitive sub-scale to participants who were 2.5–2.99 years old as a measure of cognitive development (*n* = 3). A subset of the participants 2.50–4.99 years old in one of the two studies were also administered the cognitive sub-scale of the Bayley-4 when time in the assessment and participant motivation allowed (*n* = 12). Raw scores from participants were transformed into age equivalent scores in months for use in analyses.

##### Derivation of mental age equivalent scores.

A key objective of this study was to determine the developmental sensitivity of this adapted cognitive flexibility measure for young children with DS. To do so, scalable age equivalent scores were necessary. To address the floor effects for the SB5-ABIQ age equivalent scores, MA estimates from the Bayley-4 cognitive sub-scale were used when participant engagement and time in the assessment allowed. In analyses with the available assessments, MA equivalents were derived from Bayley cognitive scores for 15 participants and SB5-ABIQ for 57 participants.

The Bayley-4 is able to capture greater variability in cognitive ability compared with the SB5-ABIQ for young children with DS. However, there remained a sizable subgroup of participants with age equivalent scores at the floor of the SB5 who did not have Bayley-4 administrations. To address this limitation, additional analyses were conducted with child participants binned in 1-year age bands for MA estimates. For participants at the SB5 floor with no Bayley-4 administration (*n* = 23), age equivalent scores were designated as within the 1-year MA band (i.e. below 24 months). The 2-year age band was defined as age equivalent scores between 25 and 35 months. Each subsequent MA band involved 12-month windows: 36–47, 48–59, 60–71, 72–83 and 84–95 months.

#### Cognitive flexibility laboratory task

##### Adapted reverse categorisation.

Child participants completed an adaptation of a reverse categorisation task, with administration adjustments to account for the needs of young children with DS. The task involved inviting child participants to sort toys according to a colour-congruent rule and then instructing them to shift to a new rule in which toys were sorted according to a colour-incongruent rule. This task was adapted from Carlson *et al*. (2004) for children with DS by modifying the instructions to reduce cognitive demands (e.g. removed size as sorting characteristic) and receptive language demands (e.g. simplified verbal instructions provided along with visual/gestural supports). Additional considerations and adjustments included increasing the salience of the differences between the two types of objects, including the use of high contrast colours between the toys to address challenges with visual acuity and the use of two different types of toys that corresponded with colours.

Colour (red versus yellow) was the key sorting dimension for the rule-based game. Children were presented with a red toy (a block) and a yellow toy (a ball), along with a red bucket and a yellow bucket. Rather than prompting children to put red toys in the red bucket, additional adaptations to the task involved affixing the buckets with pictures of easily identifiable US-based cultural references commonly associated with the sorting colours. The references selected were commonly available food condiments in the USA that are very familiar to children; one bucket was affixed with a picture of a red ketchup bottle (label was generic in nature), and the other was affixed with a picture of a yellow mustard bottle (label appearance was generic in nature). To reduce receptive language demands, condiment names were used in instructions rather than colour names. This is because identifying objects (nouns) requires less advanced receptive language understanding than identifying object properties (adjectives; Mintz & Gleitman 2002). In the colour-congruent phase, children were instructed to sort the ‘ketchup’ (with gestures referring to the red blocks) into the red ‘ketchup’ bucket and ‘mustard’ (with gestures referring to the yellow balls) into the yellow ‘mustard’ bucket.

Examiners administered two teaching trials to each participant. For children who correctly answered on the first teaching trial, the examiner affirmed the child’s correct response with ‘That’s right! The “ketchup” goes into the “ketchup” bucket.’ If a child incorrectly sorted a toy on the teaching trials, the examiner responded with a corrective prompt and demonstration of the correct answer, ‘That’s not quite right, the “ketchup” goes into the “ketchup” bucket like this. Now you try!’ Participants could receive teaching prompts up to two times per individual teaching trial. The trial was re-administered with the item label and instructions, ‘This is “ketchup,” where does it go?’ Examiners then moved on to administer the test trials with the same label and instructions on every trial. Children completed 10 trials following a colour-congruent prompt, designated as ‘pre-switch’ trials.

After the pre-switch trials, a new colour-incongruent rule was introduced by saying, ‘Now we are going to play the *silly* game! I want you to put the mustard in the ketchup bucket, and the ketchup in the mustard bucket. Let’s try one!’ Children received two teaching trials for the silly game, and the examiner provided feedback in the same structure as with the pre-switch trials. The task was ended when (1) the child sorted each toy for all 20 test trials, (2) the child gave three consecutive incorrect responses, (3) the child was non-responsive to three consecutive trials or (4) the child did not adopt the first sorting rule during the pre-switch phase (e.g. the child demonstrated 50% accuracy or lower on pre-switch trials). Scores between 0 and 10 points were possible for each trial (pre-switch and post-switch).

Participants who sorted at least 6 of 10 trials correctly in the pre-switch trials were included in analyses for the post-switch trials. Performances on post-switch trials were categorised by accuracy, with designations at the ceiling, floor or ‘emerging’. The ceiling was defined as scoring a 10 of 10 on post-switch trials; emerging flexibility was defined as a score of 1 to 9 on post-switch trials; children at the floor of the post-switch trials scored 0 of 10.

The task was scored by the examiner *in vivo* for child behaviour (off-task behaviour, inattention) and through behavioural coding software with Noldus Observer XT (Noldus Information Technology 2013). Inter-rater reliability was established with 30% of videos, with strong inter-rater agreement (frequency codes kappa = 0.73). Coders observed the accuracy of sorting and the number of times participants either changed their answer from a correct sort or self-corrected to sort the toy by the rule.

### Analysis plan

The analytic plan involved the evaluation of the feasibility, developmental sensitivity and reliability of the ARC measure of cognitive flexibility. Task feasibility was evaluated by identifying the percentage of children in the age range of interest able to provide at least one correct sorting response on the pre-switch trials, with an a priori rate designated at 80%. Descriptive statistics were also generated to characterise child behaviour and responses for participants who demonstrated behaviours that may have impacted the length of the task but did not impact the participant’s opportunity to complete the task (e.g. refusal, inattention or fatigue).

Pirate plots were generated to visualise the distribution of post-switch performances by using CA and MA as categorical variables separated by year. Preliminary evaluation of test–retest reliability was conducted with a two-way random-effects intraclass correlation coefficient (ICC) for the subgroup of participants who completed the assessment again 2 weeks later. Good reliability criteria of ICC > 0.75 were set a priori, with values 0.50 to 0.75 indicating moderate reliability (Koo & Li 2016; Schworer *et al*. 2023).

## Results

### Preliminary analyses

No significant cross-site differences were observed in post-switch trial performance, *F*_2, 69_ = 1.31, *P* = 0.28. No significant differences in post-switch performance were observed based on use versus non-use of COVID-19 precautions, *F*_1, 70_ = 0.082, *P* = 0.78.

### Feasibility

Feasibility was first evaluated by examining the number of participants able to correctly sort at least one item on the pre-switch trials. There were 65 participants (90.3%) who met this criterion, and thus, the task met the 80% feasibility threshold in this sample. Scores not at the floor on the pre-switch trials ranged from 1 to 10, with 40 participants (55.6%) scoring 10 out of 10 points. Fifty-five participants (76.4%) met the threshold to continue to the post-switch trials by scoring at least 6 in the pre-switch trials. The distribution of performances differed for the post-switch trials, where 37 participants (51.4%) scored at the floor (a score of 0). Once again, performances ranged from 1 to 10 for scores not at the floor, with 19 participants (26.4%) scoring a 10 out of 10 (Table 2).

Feasibility was also evaluated by analysing examiner observation of child behaviour throughout task administration. There were 10 participants (13.9%) who demonstrated a range of off-task behaviours during the administration (e.g. throwing), but these behaviours did not prevent task completion. Most participants who demonstrated off-task behaviours had a CA of 5 years or younger, and the majority had an MA estimate in the 1-year age range. One child with an MA in the 4-year age range demonstrated off-task behaviours. There were 13 participants (18.1%) who initially complied with task participation and then demonstrated refusal during task administration, thus ending the task prematurely. Of the participants who refused the task, five participants successfully completed the pre-switch trials before refusal behaviour ended the task with the post-switch trials. The majority of the participants who refused had a CA of 3 to 5 years, although one 6 year old also demonstrated refusal, and the majority had an MA in the 1- to 2-year age range, although one child with an MA in the 3-year-old age range refused the task as well. Finally, there were four participants (5.6%) who demonstrated inattention during the task, ranging in age from 2 to 7 years, all of whom had an MA below 3 years. Overall, off-task behaviours were most likely to be observed in participants with MA estimates under the age of 3 years and only a few cases (*n* = 2) of participants demonstrating these behaviours when older than 5 years chronologically.

Several participants demonstrated either change-from-correct or self-correction responses. Seven participants (11.1%) had one self-correct during the trials, and seven participants (11.1%) had two, three, or four self-correction responses. A similar number of participants had responses where they changed their answer from the correct bucket to the incorrect bucket. This included six participants (9.5%) who changed from the correct answer one time and four participants (6.4%) who changed from correct either two or three times.

### Performances by chronological age and mental age bands

**Figure 1.**
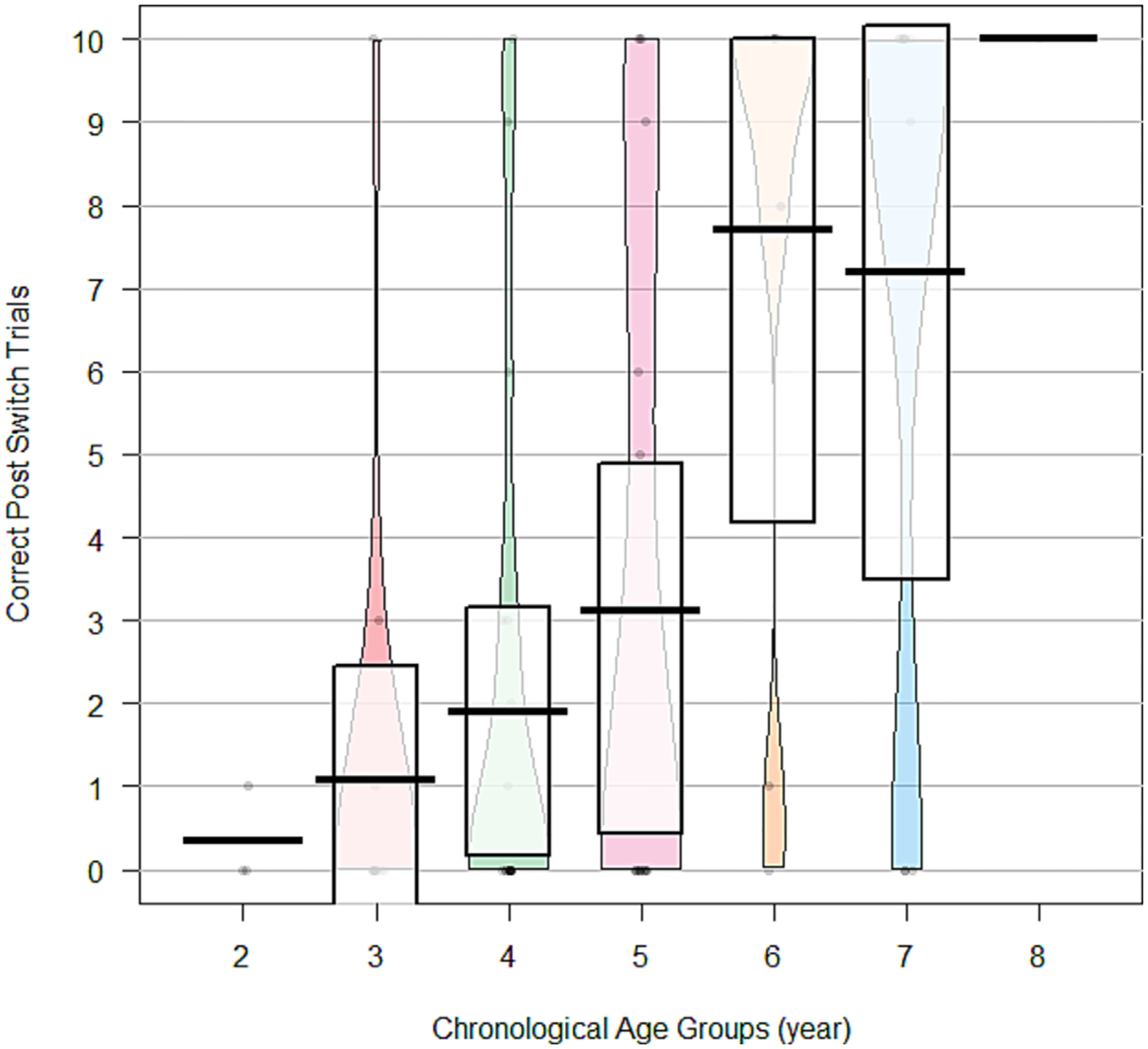
Visualisation of post-switch correct trials by chronological age group. This pirate plot shows the accuracy of participants in the post-switch trials by their chronological age year. Pirate plots show measures of central tendency and capture the distribution of data across each of the age bands by plotting observed individual scores.

A significant correlation was observed between child CA (as a continuous variable) and raw number of post-switch trials correct, *r*(70) = 0.58, *P* < 0.0001. Post-switch performances were then divided into three groups to examine performance patterns by CA 1-year age bands. Participants were designated as demonstrating post-switch performances at the floor (score of 0 out of 10; *n* = 37), between one and nine items correct (*n* = 16) or at the ceiling (score of 10 out of 10; *n* = 19). To visualise participant performance by CA on post-switch trials, a pirate plot was generated (Fig. 1). Performance improved dramatically with increasing CA (Table 3 and Fig. 1), with 60–70% of participants under the age of 5 years scoring at the floor for post-switch items, but post-switch floor decreased dramatically in children over 5 years. A similar trend was observed at the ceiling of the measure, with less than 10% of participants under the age of 5 scoring at the ceiling, but approximately 60% of children over age 6 doing so.

**Figure 2.**
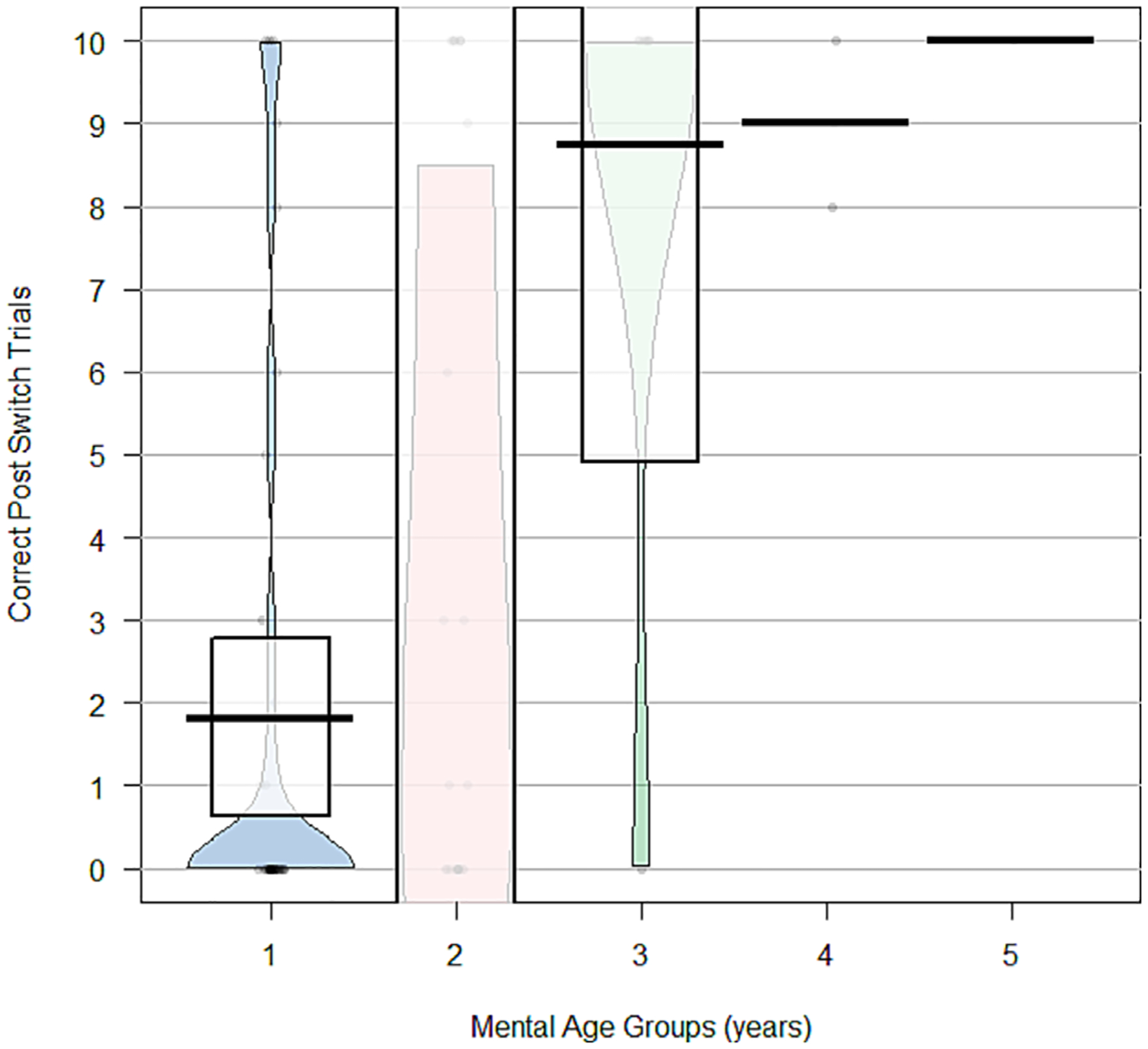
Visualisation of post-switch correct trials by mental age groups of 1 year. This pirate plot shows the accuracy of participants in the post-switch trials by their mental age year. The mental age estimate was derived from scores using the Bayley cognitive (*n* = 15) and the SB5-ABIQ (*n* = 57). Pirate plots show measures of central tendency and capture the distribution of data across each of the age bands by plotting observed individual scores.

A significant correlation was also observed between child MA (as a continuous variable, with some degree of floor effects at the 24-month age equivalent) and raw number of post-switch trials correct, *r*(70) = 0.56, *P* < 0.0001. For those in the 1-year MA band, 69.4% scored at the floor and less than 10% of participants were at the ceiling (8.3%). At an MA of 2 years, 45.8% of participants were at the floor and 29.2% of participants scored at the ceiling (Table 4). Data visualisation for the number of correct post-switch trials (Fig. 2) demonstrates that the greatest variability in performances was observed within the 2-year-old MA band, with increasing accuracy across the older age bands (Table 4 and Fig. 2).

### Test–retest reliability

A subgroup of participants (*n* = 28) completed the task again 2 weeks after their initial participation. Two-way random-effects ICCs indicated good reliability between assessments for both post-switch accuracy (ICC (A,1) = 0.81; *F*_26, 26_ = 9.42, *P* < 0.001) and total accuracy (ICC (A,1) = 0.78; *F*_26, 26_ = 8.05, *P* < 0.001). Those who completed the retest administration were similar in CA to those who did not *t*(43.85) = −1.16, *P* = 0.25 (equal variances not assumed). We note, however, that the mean age of those who engaged in the test–retest trials was 5.49 years (SD = 1.76), whereas the mean age of those who did not was 5.05 years (SD = 1.24). Therefore, preliminary evidence for test–retest reliability should be narrowly interpreted within an older CA range.

## Discussion

This study characterised the performances of children with DS on an adapted version of a reverse categorisation task, an early childhood cognitive flexibility paradigm (Carlson *et al*. 2004; Carlson 2005). At present, no measures of early childhood cognitive flexibility have been rigorously evaluated for use in young children with DS. Results provide preliminary evidence that the ARC task is developmentally sensitive and feasible for assessment of young children with DS, with preliminary evidence for test–retest reliability.

The ARC task was selected for evaluation because it accounts for factors that might confound the interpretation of performance in young children with DS. This task involved no expressive language demands, minimal receptive language demands and minimal motor planning demands. As such, this ARC task can be used to evaluate cognitive flexibility in children with minimal expressive language skills as well as those with more advanced language use, allowing for representative samples in DS studies and reduced task impurity (Willoughby & Hudson 2021). Other adaptations of this task were made to increase interest level by using familiar US-based cultural referents and heightening the contrast between the two items through the selection of object colours and shapes. Analyses from this investigation support the utility of this task for young children with DS.

### Feasibility

Results demonstrated that this task had adequate feasibility because over 80% of participants could achieve a score during the pre-switch trials and did not display behaviours precluding meaningful participation in the task. When refusal behaviour did end the task prematurely, it may have been the result of challenge with the task or the timing of the task within the full assessment battery. Thus, ARC can be attempted in a wide range of early childhood for children with DS and be engaging to a majority of participants in this developmental range.

### Developmental sensitivity

As observed in cognitive flexibility tasks administered to children without disabilities (Carlson 2005), this measure tracked quite closely with both CA and MA. Raw scores for performance on post-switch trials, the primary outcome for this measure, were significantly positively correlated with both CA and MA. In addition, when post-switch performances were divided into three categories: floor (a score of 0), ceiling (a score of 10) and emerging (scores between 1 and 9), developmentally contingent changes were observed across the 1-year age bands established for both CA and MA.

Based on observed performances by CA bands, only one participant under the age of 4 years scored at the ceiling on the post-switch trials, and only one scored in the emerging category. The majority of participants under 4 years scored at the floor, and participants began to demonstrate more variability across the three categories of performance between ages 4 and 5 years chronologically. A small number of participants between the ages of 6 and 7 years continued to demonstrate performances at the floor, while the remaining participants scored in the emerging or ceiling categories. These findings suggest that assessment on this task can be informative within the present CA range, depending on the goals of future study designs. If description of performances is of interest, for example, to identify correlates of performances, then children in the 4- to 5-year age range may be in the ideal CA window for use of this measure. Alternatively, if cognitive flexibility is a target for intervention or treatment, and this measure is used as an outcome measure, children in the younger CA bands may be within the ideal window for this measure.

Based on observed performances for MA bands (Table 4), the recommended MA range for this task appears to be within the 1- to 3-year MA range, which captures the majority of participants in this study. By an MA of 3 years and older, most participants scored at the ceiling for this task. However, it is notable that even in the 3- and 4-year MA bands, a small number of participants continued to show scores at either the floor or the emerging performance level.

Interpretations of these older MA performances may be nuanced and could possibly reflect socially playful and knowingly incorrect responses, as observed with the use of social strategies during instrumental tasks in young children with DS (Wishart 1996; Kasari & Freeman 2001; Fidler *et al*. 2005; Cebula *et al*. 2010).

### Limitations

This study contributes to the effort to evaluate measures of critical developmental outcomes that are of relevance to individuals with DS and ID. In the context of the findings reported, several issues should be taken into consideration. First, the sample included in this investigation was largely White and of higher income than the general population. Future work should aim to include participants from a variety of ethnic, racial and economic backgrounds to increase the generalisability of ARC for cognitive flexibility in this population. Future work should also consider the use of cultural-specific references for administration outside of the USA. This sample of children with DS may have also overrepresented children with a congenital heart defect (CHD). Additional work should examine the interaction between CHD and child performances. This investigation is also limited in its sample size relative to other psychometric investigations (Miyake *et al*. 2000; Carlson 2005); however, it is a relatively large sample size for a low incidence neurogenetic condition, like DS. Data collection for this project began just prior to the onset of the COVID-19 pandemic, and examiners took precautions that were aligned with the requirements at each site. No statistically significant differences were observed based on the use or non-use of these safety precautions; however, it is unclear how participation during the complex period when COVID-19 predominated may have influenced child performances in other ways.

An additional issue to consider in the interpretation of findings relates to the MA bands. First, it is noted that age equivalent scores are an imperfect estimate of overall functioning, as they represent the median score from a norming sample of a particular age group and are not interval or ratio scales of measurement (Conrad 2018). In addition, although the SB5-ABIQ is increasingly selected as a common data element for measuring IQ in this population, it is difficult to find one cognitive assessment that is adequately able to capture the abilities of children with DS in the CA range for this study. Some children were administered the Bayley-4 cognitive domain to provide supplementary information beyond the floor age equivalent score generated by the SB5-ABIQ, but many were not. Future work should seek to identify ways to capture cognitive status without the use of multiple measures and floor effects.

Even when considering these study limitations, the ARC task demonstrated feasibility and developmental sensitivity in young children with DS. Preliminary evidence for test–retest reliability was observed as well. The present evaluation of this measure of cognitive flexibility demonstrates its potential utility as an outcome measure for future developmental research and potential treatment trials that target the development of cognitive flexibility throughout early childhood in children with DS.

## Figures and Tables

**Table 1:** Demographic information

Child variable	%(*n*)
% male (*n* = 3 missing)	44.9 (31)
Child chronological age (years; SD)	5.22 (1.47)
Child developmental age (years; SD)	2.38 (0.84)
Race (*n* = 5 missing)	
Asian American	4.5 (3)
Black/African American	3.0 (2)
White	85.1 (57)
Other	7.5 (5)
Ethnicity (*n* = 9 missing)	
Hispanic	14.3 (9)
Not Hispanic	85.7 (54)
DS type (*n* = 4 missing)	
Trisomy 21	89.7 (61)
Mosaicism	1.5 (1)
Translocation	4.4 (3)
Not sure	4.4 (3)
Premature birth (% yes; *n* = 4 missing)	25.0 (17)
Congenital heart defects (% yes; *n* = 4 missing)	72.1 (49)
Caregiver variable	%(*n*)
Primary caregiver age (years; mean/SD; *n* = 4 missing)	40.65 (6.26)
% primary caregiver education at least 1 year of college/tech training (n; *n* = 6 missing)	97.0 (64)
% annual income (*n*; *n* = 5 missing)	
Below $50 000	10.4 (7)
$50 000–100 000	23.9 (l6)
Above $100 000	62.7 (42)
Did not wish to provide	3.0 (2)

DS, Down syndrome; SD, standard deviation.

**Table 2:** Observed behaviours split by performance and task phase

Task phase	Floor %(*n*)			Emerging %(*n*)			Ceiling %(*n*)		
Pre-switch		9.7 (7)			34.8 (25)			55.6 (40)	
	**Other off-task behaviours (*n*)**	**Inattention to task (*n*)**	**Refusal to participate (*n*)**	**Other off-task behaviours (*n*)**	**Inattention to task (*n*)**	**Refusal to participate (*n*)**	**Other off-task behaviours (*n*)**	**Inattention to task (*n*)**	**Refusal to participate (*n*)**
	1	0	6	5	2	2	4	2	5
Post-switch		51.4 (37)			22.2 (16)			26.4 (19)	
	**Other off-task behaviours (*n*)**	**Inattention to task (*n*)**	**Refusal to participate (*n*)**	**Other off-task behaviours (*n*)**	**Inattention to task (*n*)**	**Refusal to participate (*n*)**	**Other off-task behaviours (*n*)**	**Inattention to task (*n*)**	**Refusal to participate (*n*)**
	8	3	12	2	1	1	0	0	0

**Table 3:** Accuracy on post-switch trials by chronological age

	Post-switch correct groups		
Chronological age groups (years)	Zero correct post-switch % (*n*)	Emerging accuracy post-switch % (*n*)	All correct post-switch % (*n*)
2.5	66.7 (2)	33.3 (1)	0 (0)
3.0	76.9 (10)	15.4 (2)	7.7 (1)
4.0	61.1 (11)	33.3 (6)	5.6 (1)
5.0	62.5 (10)	18.8 (3)	18.8 (3)
6.0	10.0 (1)	30.0 (3)	60.0 (6)
7.0	27.3 (3)	9.1 (1)	63.6 (7)
8.0	0 (0)	0 (0)	100 (1)

**Table 4:** Accuracy on post-switch trials by mental age

Mental age groups (years)	Percent post-switch correct groups		
	Percent zero correct post-switch (*n*)	Emerging accuracy post-switch (*n*)	Percent all correct post-switch (*n*)
1.0	69.4 (25)	22.2 (8)	8.3 (3)
2.0	45.8 (11)	25.0 (6)	29.2 (7)
3.0	12.5 (1)	0 (0)	87.5 (7)
4.0	0 (0)	66.7 (2)	33.3 (1)
5.0	0 (0)	0 (0)	100 (1)

## Data Availability

Data are available upon request from the corresponding author.
